# Deformation and Fracture Behaviour of Heterostructure Mn8/SS400 Bimetal Composite

**DOI:** 10.3390/ma18040758

**Published:** 2025-02-08

**Authors:** Shengnan Yuan, Cunlong Zhou, Haibo Xie, Mengyuan Ren, Fei Lin, Xiaojun Liang, Xing Zhao, Hongbin Li, Sihai Jiao, Zhengyi Jiang

**Affiliations:** 1 School of Mechanical, Materials, Mechatronic and Biomedical Engineering, University of Wollongong, Wollongong, NSW 2522, Australia; sy600@uowmail.edu.au (S.Y.); xie@uow.edu.au (H.X.); mr138@uowmail.edu.au (M.R.); flin@uow.edu.au (F.L.); 2 School of Mechanical Engineering, Taiyuan University of Science and Technology, Taiyuan 030024, China; zcunlong@tyust.edu.cn; 3 Northwest Institute for Nonferrous Metal Research, Xi’an 710016, China; 4 Baosteel Research Institute (R&D Centre), Baoshan Iron & Steel Co., Ltd., Shanghai 200431, China; liangxiaojun@baosteel.com (X.L.); xingzhao@baosteel.com (X.Z.); lihongbin@baosteel.com (H.L.); shjiao@baosteel.com (S.J.)

**Keywords:** bimetal composite, deformation, plastic instability strain, fracture mechanism

## Abstract

This study examines the deformation behaviour and fracture mechanisms of bimetal composites (BCs) composed of high-carbon medium-manganese steel (Mn8) and low-carbon steel (SS400), fabricated through hot roll bonding. The research highlights the effect of varying thickness ratios on the mechanical properties of Mn8/SS400 BCs. The microstructure and interfacial characteristics were analysed using scanning electron microscopy (SEM), revealing a well-bonded and defect-free interface with distinct elemental distributions. Tensile and bending tests were conducted to evaluate the composites’ mechanical performance, highlighting the synergistic effects of Mn8’s high strain hardening capacity and SS400’s ductility. Mathematical models, including the rule of mixtures (ROM) and the long-wavelength approach (LWA), were employed to predict the tensile strength and plastic instability strain (PIS), with experimental results showing deviations due to interfacial strengthening mechanisms and dislocation pile-ups. The findings provide insights into the interplay between layer thickness ratios, interfacial properties, and strain hardening, offering valuable guidance for optimising the design and industrial-scale production of Mn8/SS400 BCs.

## 1. Introduction

The rapid advancement of technology and industrial progress has driven a growing demand for materials with exceptional and multifunctional performance across various industries. This demand highlights the need to develop materials that exhibit a combination of outstanding properties designed to meet the evolving requirements of industrial production [1]. By combining materials with complementary properties, innovative composite materials can be designed to fully utilise the unique strengths of each component [2]. As a result, substantial research efforts have been directed toward exploring novel approaches and advanced technologies for fabricating composites that deliver superior mechanical performance [3,4,5]. Extensive research has been conducted on laminated composites formed from various material combinations, including steel-bronze [6], Cu-steel [7,8,9], Ti-steel [10], and stainless steel-carbon steel [11]. The majority of existing research on BCs has been dedicated to their fabrication [12,13] and analysis of interface bonding properties [14,15]. In contrast, limited attention has been given to exploring the synergistic deformation behaviour of these materials.

For laminated composites and BCs, the correlation between the mechanical behaviour and its constituent materials is a critical factor in predicting composite performance. Some experimental results and research have demonstrated that the strength and stress–strain behaviour of BCs can be effectively estimated using the rule of mixtures (ROM), expressed as $\sigma =V_{1}\sigma_{1}+V_{2}\sigma_{2}$, where $V_{1}$ and $V_{2}$ represent the volume fractions of the two components, and $\sigma_{1}$ and $\sigma_{2}$ denote their respective strengths. However, the ROM considers only the volume fraction and strength of the two components, overlooking the impact of the interface and mechanical incompatibility of layers on the deformation behaviour of BCs. Zhao et al. [16] observed a significant discrepancy between ROM-predicted curves and experimental results during uniaxial tensile tests on coarse-grained (CG) and nano-grained layered pure copper. They attributed this divergence to interface strain gradient effects, back stress contributions, and strengthening mechanisms induced by non-uniform deformation. Wang et al. [17] utilised laminated composites consisting of CG pure copper and nanostructured Cu-Zn alloy layers with different volume fractions and conducted uniaxial tensile and loading-unloading-reloading tests. The experimental results exceeded ROM predictions due to enhanced back stress, strain gradient effects near interfaces, and synergistic deformation mechanisms between the layers. In addition, the pronounced differences in the ductility of individual components can lead to stress concentrations at the interfaces and premature failure of less ductile layers [18]. Furthermore, the significant disparity in strain hardening properties between the constituent layers, along with the additional strengthening effects introduced by the interface, are critical factors to consider when calculating the strength of BCs [19,20,21].

PIS is a critical parameter often used as a criterion to evaluate the plasticity of materials. It denotes the initiation of localised deformation, where uniform plastic flow transitions into strain concentration, ultimately resulting in necking or failure. Its significance is underscored by its strong correlation with fundamental material properties, including strain-hardening capacity, ductility, and microstructural features such as the grain size, dislocation density, and phase distribution. This relationship was also widely investigated in a variety of materials [22]. Llorca et al. [23] developed a finite element analysis approach combined with a Weibull statistical model to predict the PIS in ductile matrices reinforced with brittle spherical particles. The model’s advantage lies in its ability to quantify the influence of reinforcement fracture on PIS with reasonable accuracy. Kubin et al. [24] utilised the physical properties of individual materials combined with strain-hardening models to predict the PIS of Al alloys/IF steel laminated composites, achieving high accuracy due to the well-characterised properties of these materials. Peng et al. [25] employed the long-wavelength prediction method combined with strain hardening models to calculate the PIS of a tin bronze/1010 steel bimetal layered composite. The prediction was found to be moderately accurate for overall strength, and the advantage of the method lies in its simplicity and applicability to layered composites with well-characterised individual components.

The fracture mode is a critical factor in defining the mechanical performance and reliability of materials. Whether the fracture is ductile, brittle, or a mixed mode, it profoundly impacts key properties such as the strength, toughness, and energy absorption capacity [26]. In a BC comprising two materials with distinct fracture modes, variations in the volume fraction of each constituent can significantly influence the overall deformation behaviour of the composite. Despite its importance, this aspect has received limited attention in existing research. Medium manganese steel, such as Mn8 steel (a high-carbon, fully austenitic medium manganese steel), has garnered significant attention in research due to its outstanding properties, including superior strength, excellent ductility, and enhanced plasticity and impact resistance facilitated by the twinning-induced plasticity (TWIP effect). These attributes make it a critical material for advancing high-strength steel technologies and investigating multifunctional mechanical behaviours [27,28,29,30]. However, its industrial application in bimetallic composites is still underexplored, especially in terms of the synergistic interaction between Mn8 and other steels with complementary properties. SS400 offers exceptional mechanical compatibility and processability, making it a suitable base material for Mn8 composite plates. Its high ductility and plasticity effectively alleviate stress concentrations when paired with Mn8’s high strength, ensuring an optimised balance between overall strength and toughness [31,32,33]. The combination of these two materials provides an excellent platform for developing high-performance BCs. This study, therefore, selects Mn8 and SS400 steels as representative materials to explore the deformation and fracture behaviour of bimetallic composites. This study systematically investigates the deformation and fracture mechanisms of Mn8/SS400 steel BCs fabricated via hot roll bonding, with a specific emphasis on the influence of thickness ratios on mechanical performance. By combining microstructural characterisation and mechanical testing methods, such as tensile and bending tests, the research offers detailed insights into the tensile strength and plastic instability strain (PIS) of the BCs. These findings are further evaluated using the rule of mixtures (ROM) and long-wavelength predictions to determine their reliability in describing complex bimetallic systems. Through this analysis, the study contributes to a deeper understanding of the deformation and fracture behaviour of Mn8/SS400 BCs and provides practical guidance for the design, large-scale production, and industrial application of bimetallic composites with optimised properties, addressing critical gaps in current knowledge.

## 2. Experimental Materials and Methods

### 2.1. Materials and Sample Preparation

**Figure 1:**
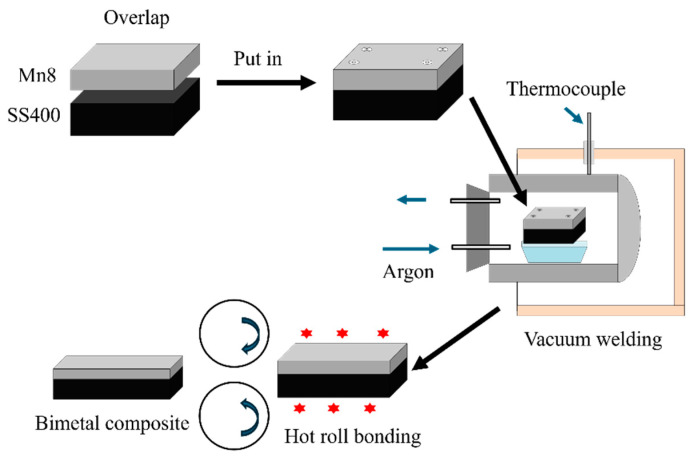
Illustration of the vacuum welding and hot roll bonding process of the Mn8/SS400 BCs.

This study utilized Mn8/SS400 BC plates, which were industrially manufactured through a hot roll bonding process by Baoshan Iron & Steel Co., Ltd. (Shanghai, China). The fabrication involved vacuum welding as a preparatory step, followed by hot roll bonding conducted at temperatures between 800 and 1100 °C. Figure 1 shows the fabrication process of the Mn8/SS400 BCs. The final composite consisted of a 3 mm Mn8 steel layer firmly bonded to a 17 mm SS400 low-carbon steel substrate. Both Mn8 and SS400 steel layers were milled to achieve the desired thickness and proportions, and samples were extracted from the as-received BC plates for microstructural analysis and mechanical property testing. The detailed chemical compositions of the Mn8 and SS400 steel layers are listed in Table 1.

### 2.2. Microstructure Characterisation

The microstructures and elemental distribution of the BC samples were analysed using a JEOL JSM-7001F scanning electron microscope (SEM) equipped with energy-dispersive spectroscopy (EDS) (Tokyo, Japan). Samples for SEM were prepared by grinding with sandpaper up to 5000 grits, followed by polishing and etching with a 7.5% Nital solution. This preparation ensured a smooth surface for accurate microstructural analysis.

### 2.3. Mechanical Testing

**Figure 2:**
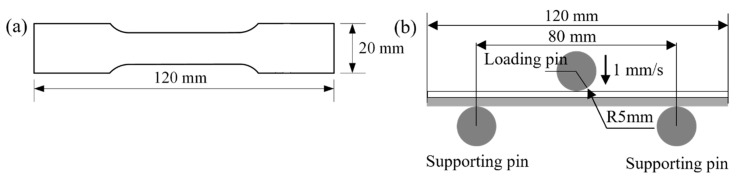
The dimensions of the specimens for mechanical testing: (**a**) tensile test samples and (**b**) three-point bending test samples (units: mm).

Tensile specimens with varying thickness ratios of Mn8 and SS400 steel, each with a total thickness of 5 mm, were prepared according to the dimensions provided in Figure 2a. The Mn8/SS400 BC samples were sectioned using a Wire Electrical Discharge Machining system (WEDM, John Hart Pty Ltd., Melbourne, Australia). The tensile samples were dimensioned in accordance with the ASTM-E8 standard, and the dimensions, along with the Mn8-to-SS400 proportion for each sample, are listed in Table 2. For evaluating the individual mechanical properties of Mn8 and SS400 steels, tensile specimens with a thickness of 2.5 mm were directly extracted from the BC by removing one layer. The tensile tests were conducted using a fatigue testing system (Instron-8801, Instron, Norwood, MA, USA) under displacement control at a constant strain rate of 6.67 × 10^−4^ s^−1^. The yield strength (YS) was determined at a 0.2% offset strain, while the ultimate tensile strength (UTS) was recorded directly from the testing machine. The true strain value at peak stress was defined as the PIS.

Bending specimens measuring 120 mm in length, 40 mm in width, and 5 mm in total thickness were fabricated using the WEDM machining process. These specimens, featuring the same relative thickness ratios of Mn8 steel to SS400 steel as the tensile test samples, were specifically prepared for bending tests. The tests were conducted on the same testing machine equipped with a three-point bending fixture comprising two supporting pins and a downward loading pin. A loading pin was applied at a press speed of 1 mm/s to ensure quasi-static bending conditions. The experimental setup and the dimensions of the samples are presented in Figure 2b. Specimens were positioned on the support pins, with a span of 80 mm between them. The loading pin was displaced to a depth of 35 mm. Bending tests were carried out under two stacking configurations: With the Mn8 steel layer arranged on the outer side or the inner side during bending. In order to ensure the reliability of the experiments, at least three replicate experiments were performed for each sample.

## 3. Results and Discussion

### 3.1. Microstructure Analysis of Mn8/SS400 BC

**Figure 3:**
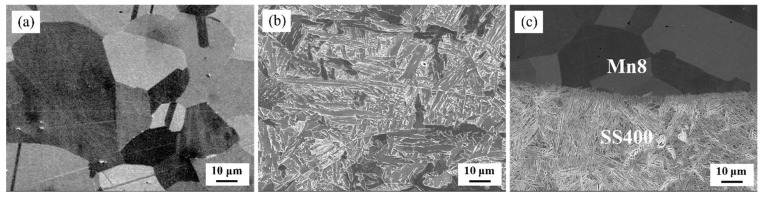
Microstructure of the as-received Mn8/SS400 bimetal composite: (**a**) Mn8; (**b**) SS400 steel; (**c**) interface.

The samples were cut from the as-received BC plates directly to observe the microstructure as shown in Figure 3. The microstructure of Mn8 steel primarily comprises austenite with a minor fraction of precipitates (Figure 3a). In contrast, the SS400 low-carbon steel microstructure consists of bainitic ferrite (BF), austenite, and martensite or retained austenite (M/RA), the latter of which presents in either thin, film-like layers or blocky morphologies as shown in Figure 3b. The film-like M/RA mixture aligns parallel to the BF laths, while blocky M/RA is found within bainitic structures. The M/RA morphology is influenced by BF structure: Film-like M/RA forms between lath-like BF, and blocky M/RA is surrounded by granular BF [31,34,35]. The Mn8 and SS400 layers in the composite exhibit distinct separation in brightness after etching, a contrast attributable to their compositional differences. This bimetallic composite demonstrates a well-defined, defect-free interface that underscores the unique microstructural characteristics of each layer as shown in Figure 3c. Due to its higher manganese content, the Mn8 layer appears darker relative to the SS400 layer, creating a sharp visual contrast that precisely delineates the boundary between the two alloys.

**Figure 4:**
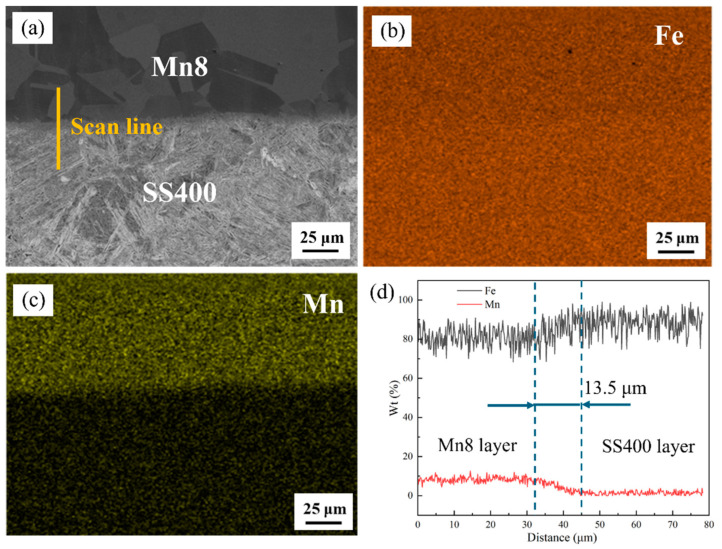
(**a**) Interfacial structure of the Mn8/SS400 BC after hot rolling; corresponding elemental distribution maps of (**b**) Fe and (**c**) Cu; and (**d**) EDS line scan profile across the interface.

Figure 4a–d show the cross-section of the fabricated Mn8/SS400 BCs as well as the element distribution after hot rolling. Figure 4b,c show the main element distribution of EDS surface scanning of the hot-rolled Mn8/SS400 BCs. The elements exhibit an uneven distribution across the layers, in which the manganese (Mn) element is mainly distributed in the Mn8 layer while the iron (Fe) element is mainly distributed in the SS400 layer. According to the EDS results, the interfaces between constituent layers are basically straight, continuous, and well bonded, and there is no sign of the formation of intermetallic compounds. During the hot rolling period, due to the compositional gradients for Fe and Mn between the parent metal and clad layer and sufficient time and temperature, the required driving force for diffusion of the elements in the BC can be easily provided. Therefore, there is a phenomenon of element diffusion between the parent metal and clad layer in the surrounding area of the bi-material interface. The Fe and Mn elements exhibit migration in opposite directions across the bi-material interface. Specifically, Mn migrates from the clad layer toward the bi-material interface and subsequently into the parent metal, while Fe moves in an opposite direction. This behaviour is inferred from the observed distribution profiles of Fe and Mn across the bi-material interface. As shown in Figure 4d, Fe and Mn elements were observed to diffuse from their respective layers across the composite interface, with diffusion distances of approximately 13.5 µm. The interfacial diffusion behaviour of elements between the steel layers is a critical factor governing the bonding quality and interfacial strength of BCs. Notably, an optimal diffusion distance of alloying elements is typically associated with superior interfacial bonding strength [36,37].

### 3.2. Tensile Behaviour of Mn8/SS400 BCs

**Figure 5:**
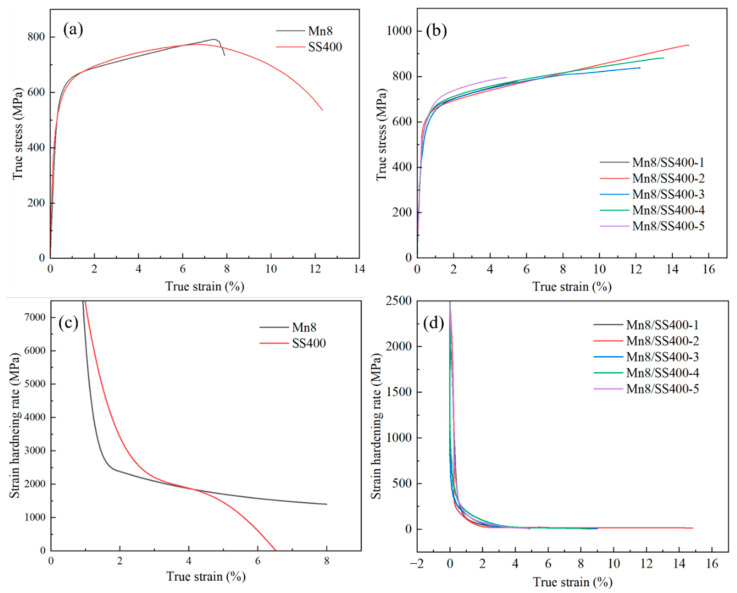
True stress–strain curves of (**a**) individual Mn8 and SS400 steel, and (**b**) Mn8/SS400 BCs; the strain hardening rate curves of (**c**) individual Mn8 and SS400 steel, and (**d**) Mn8/SS400 BCs.

To investigate the strength and work-hardening behaviour of the Mn8/SS400 BC, a standard quasi-static uniaxial tensile test was conducted, from which the engineering stress–strain curves were directly acquired. The true stress–strain curves for Mn8 and SS400 steel were derived using Equations (1) and (2) [8], as illustrated in Figure 5a. The Mn8 steel exhibits a true YS of 597.96 MPa and a UTS of 792.68 MPa, while the SS400 low carbon steel shows a YS of 542.70 MPa and a UTS of 774.19 MPa. Mn8 steel exhibits a slightly higher degree of uniform plastic deformation; however, it transitions almost immediately to fracture without experiencing a nonuniform deformation phase. In contrast, SS400 steel undergoes a well-defined phase of nonuniform deformation, characterised by necking, prior to reaching complete fracture, highlighting its greater ductility.

(1) $$\varepsilon =\ln\left(1+\varepsilon_{E}\right)$$

(2) $$\sigma =\sigma_{E}\left(1+\varepsilon_{E}\right)$$

Figure 5b shows the TSS curves of the Mn8/SS400 BCs, and Table 3 shows the corresponding YS, UTS, and UL values. The mechanical properties of Mn8/SS400 bimetal composites, particularly UTS and UL, are strongly influenced by the thickness ratio of the two layers. Balanced thickness ratios, such as 1:2 or 2:1, result in significantly higher UTS and UL, whereas extreme ratios, such as 1:5 or 5:1, lead to notable reductions in both properties. This phenomenon is closely related to the interplay between lattice mismatch, interfacial strain, and the behaviour of geometrically necessary dislocations (GNDs).

**Figure 6:**
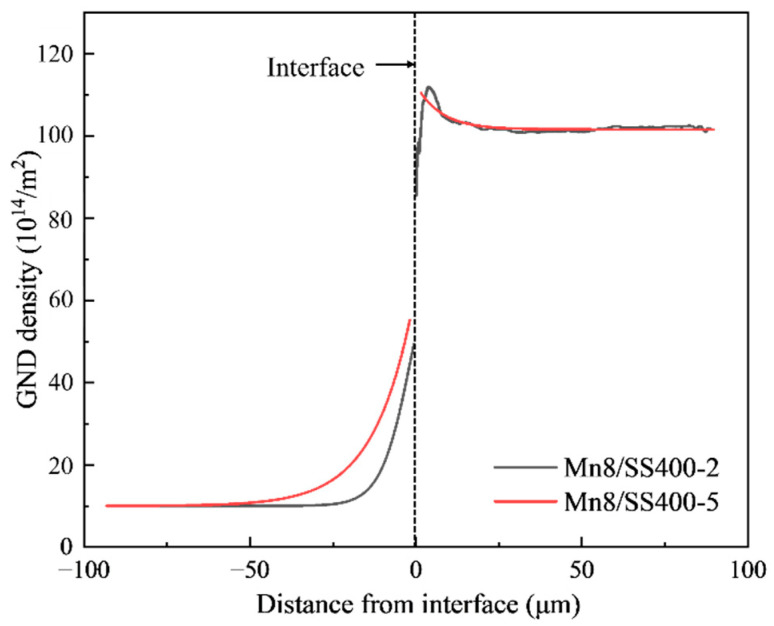
Averaged GND density as a function of equivalent distance from the interface for Mn8/SS400-2 and Mn8/SS400-5 samples (Negative values on the x-axis correspond to the Mn8 layer, while positive values represent the SS400 steel layer).

The intrinsic differences in crystal structures and structural distinctions between Mn8 and SS400 contribute to lattice constant disparity. The high manganese and carbon content in Mn8 expands its lattice, resulting in a larger lattice constant compared to SS400, which is a low-carbon steel with a more compact lattice. The lattice mismatch parameter (δ) can be expressed as [38]:

(3) $$\delta =\frac{2\left|a_{Mn8}-a_{SS400}\right|}{a_{Mn8}+a_{SS400}}\times 100\%$$

where $a_{Mn8}$ and $a_{SS400}$ are the lattice constants of the Mn8 and SS400 steel, respectively. The lattice mismatch between Mn8 (FCC structure) and SS400 (BCC structure), calculated to be 23%, creates substantial strain gradients at the interface due to differences in atomic packing density and lattice constants. These gradients drive the formation of GNDs, which accommodate the misfit strain. The average GND density along the direction perpendicular to the interface was counted for the Mn8/SS400-2 and Mn8/SS400-5 BCs, as illustrated in Figure 6. Clearly, the GND density has a higher level in the layer of SS400 steel and interface, and the level close to the interface of the Mn8/SS400-5 is higher than the Mn8/SS400-2, and its distribution is also inhomogeneous which is similar to the research of Bay et al. [39]. However, the effectiveness of GNDs in relieving strain is highly dependent on the layer thickness ratio.

When the thickness ratio is balanced (1:1, 1:2, and 2:1), the applied load is distributed more evenly across the two layers, leading to a more uniform stress and strain distribution. This balanced mechanical behaviour minimises localised stress concentrations and enhances strain compatibility at the interface. The lattice mismatch-induced strain is effectively accommodated by a well-distributed network of GNDs, reducing dislocation pileups and enabling efficient load transfer between the layers. As a result, the composite exhibits higher UTS and UL, reflecting improved interfacial bonding and deformation compatibility. In contrast, extreme thickness ratios (1:5 and 5:1) cause significant mechanical asymmetry between the layers, resulting in amplified strain gradients and uneven stress distribution. The thinner layer, whether Mn8 or SS400, is unable to effectively support the load, leading to disproportionate deformation and localised stress concentrations at the interface. These extreme conditions exacerbate GND accumulation in specific regions (Figure 6), forming dislocation pileups that act as stress concentrators. The reduced efficiency of strain accommodation and load transfer under these conditions leads to lower UTS and UL. Moreover, the thinner layer is more susceptible to premature plastic deformation or failure, further degrading the composite’s mechanical performance.

The strain hardening rates (SHR) of Mn8, SS400, and Mn8/SS400 BCs are presented in Figure 5c,d, derived from the direct differentiation of the TSS curves. The SHR of both steels maintains a gentle descent during the whole uniform plastic deformation, and the decline rate of SS400 is less than the Mn8. Additionally, Mn8 steel maintains its hardening capacity over a broader range of strain, which helps sustain uniform deformation and delay necking or failure. In contrast, SS400 reaches a near-zero strain hardening rate at a lower strain level, transitioning earlier to localised deformation (necking), which leads to nonuniform deformation and eventual fracture. This highlights the superior strain hardening behaviour of Mn8 steel over a wider deformation range, while SS400 demonstrates a quicker transition to ductile failure. The SHR of BCs shows relatively minimal variation across different thickness ratios, but slight differences are observed at specific ratios (Figure 5d). At extreme thickness ratios of 1:5 and 5:1, the SHR is at its lowest, reflecting a reduced strain hardening capacity. Conversely, the SHR increased at intermediate thickness ratios of 1:2 and 2:1, with the decline rate slowing as strain increases. This trend suggests that a moderate thickness ratio optimally enhances the strain hardening effect, while extreme thickness ratios weaken it.

### 3.3. Factors Influencing ROM Prediction Deviation

**Figure 7:**
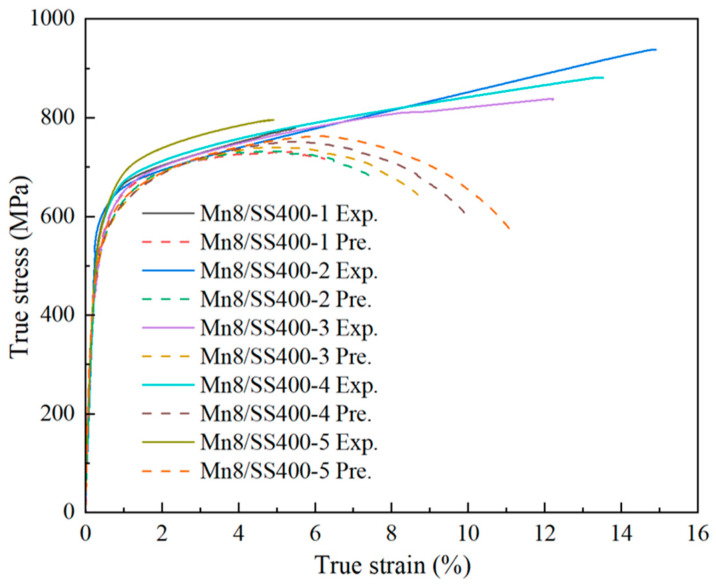
Comparison of experimental true stress–strain curves (solid lines) and ROM predictions (dashed lines).

The mechanical behaviour of the composite is strongly influenced by the properties of its constituents, making this relationship a critical topic of investigation. The ROM is widely regarded as an effective approach for predicting composite mechanical performance. In this study, the ROM was employed to systematically analyse the stress–strain behaviour of Mn8/SS400 BCs by varying the thickness ratio of Mn8 to SS400. The strengths of the individual constituents were directly measured from samples cut from the Mn8/SS400 composite. Notably, Mn8 and SS400 exhibit distinct strength and deformation characteristics when tested individually. These differences highlight the competitive and synergistic deformation behaviours within the composite. Figure 7 compares the experimental TSS curves of Mn8/SS400 BCs with predictions based on the ROM for different thickness ratios, calculated using Equation (4):

(4) $$\sigma_{c}=\;\sigma_{Mn8}V_{Mn8}+\sigma_{SS400}V_{SS400}$$

where $\sigma_{c}$ represents the composite strength, $V_{Mn8}$ and $V_{SS400}$ are the volume fractions, and $\sigma_{Mn8}$ and $\sigma_{SS400}$ are the stresses of the Mn8 and SS400, respectively. Since the ROM TSS curves were directly calculated from the stress–strain curves of the individual Mn8 and SS400 steel, the PIS is closely aligning with that of SS400. However, a significant difference in PIS between the two materials is observed. A deviation is evident between the experimental and ROM-predicted TSS curves, with experimental values generally exceeding predictions, particularly at strain values exceeding 5%. This discrepancy arises from the ROM’s limitations in accounting for non-linear deformation behaviours, strain hardening, and interfacial effects. At higher strains, complex interactions such as dislocation accumulation and interfacial bonding introduce additional strengthening mechanisms that are beyond the scope of the ROM’s linear assumptions. These mechanisms can be attributed to the accumulation of GNDs, which accommodate strain gradients, and the formation of an interfacial diffusion layer, which reduces atomic-scale incompatibilities and enhances load transfer efficiency. The pronounced discrepancy in the Mn8/SS400-2 and Mn8/SS400-4 samples highlight that their configurations are most favourable for promoting interfacial strengthening mechanisms, as balanced thickness ratios (e.g., 1:2 or 2:1) allow for more uniform strain distribution and effective GND accommodation, while extreme thickness ratios exacerbate strain gradients and reduce the efficiency of load transfer. This observation warrants further investigation to uncover the underlying reasons.

**Figure 8:**
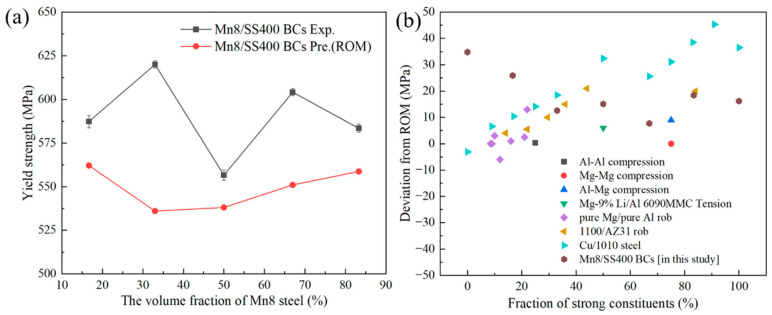
(**a**) The YS obtained by experiment and predicted by ROM versus the volume fraction of Mn8. Exp. and Pre. refer to the experimental and predicted YS, respectively, and (**b**) the deviation between the experimental YS and the ROM predictions in references [8,19,41,43,48,49].

The tensile deformation and fracture behaviour of Mn8/SS400 BCs involve intricate interactions influenced by both the heterogeneous interface and the distinct deformation characteristics of each component. To provide a comprehensive understanding, the deformation behaviour of Mn8/SS400 BCs will be systematically examined with a focus on strength, plasticity, and fracture mechanisms. Numerous studies have highlighted the effectiveness of the ROM in predicting the strength of BCs [40,41,42,43]. However, as shown in Figure 8a, the experimentally measured UTS of Mn8/SS400 BCs exceeds the values predicted by the ROM, particularly for configurations Mn8/SS400-2 and 4. This deviation can be attributed to the synergistic effects at the interface, as highlighted in previous studies [7,8,25]. These effects stem from significant stress gradients and the accumulation of GNDs at the interface [44], which generate additional back stress and enhance the composite’s overall mechanical performance. The interaction between accumulated dislocations and the interface plays a crucial role in strengthening the composite by contributing to strain transfer and stress redistribution across the layers. Moderate thickness ratios (e.g., 1:1, 1:2, and 2:1) further optimise deformation compatibility between the Mn8 and SS400 layers. These balanced thickness configurations delay localised failure, maximise the efficiency of interfacial strengthening mechanisms, and improve overall tensile properties. Interestingly, the experimental stress–strain curve of Mn8/SS400 BCs deviates significantly from the ROM-predicted behaviour, showing no distinct non-uniform deformation phase. Instead, the composite transitions directly from uniform deformation to fracture, closely aligned with the behaviour of single Mn8 steel. This response is predominantly governed by the high strength and low ductility of the Mn8 layer, which fractures prematurely and inhibits the development of necking in the SS400 layer. Furthermore, interfacial effects, including strain incompatibility and dislocation pileups, constrain the plastic deformation of the SS400 layer, limiting its ability to exhibit a non-uniform deformation phase, as reported in Mg-Nb [44] and Ti/Al [45] layered composites. These combined mechanisms underscore the complex interplay of layer properties and interface effects that drive the deviation from ROM predictions.

To enhance the credibility of the findings, this study compares the observed deviations with those reported in the prior literature (Figure 8b). While most experimental data exhibited higher YS compared to the predictions from ROM, supporting the validity of interface strengthening, certain outliers were identified. The causes of these discrepancies include (1) stress intensification at the interface, leading to residual stresses and plastic constraint effects during deformation caused by mismatched mechanical properties, such as Poisson’s ratio [46]; (2) the insufficient bonding strength can lead to interfacial debonding under stress, reducing the yield strength [43]; (3) the formation of brittle intermetallic phases can weaken the interface and lead to premature failure of the BCs [47].

### 3.4. Plastic Instability Behaviour of the Mn8/SS400 BCs

The PIS is a pivotal parameter for assessing material plasticity. The plastic instability criterion in uniaxial tensile experiments is defined as follows [25]:

(5) S·dF−F·dS=dP<0

where S is the cross-sectional area (CSA), F is the applied stress, dS represents the reduction in CSA due to plastic deformation, dF reflects the incremental strengthening, and dP indicates the change in total load-bearing capacity. This condition implies that instability arises when the loss of load-bearing capacity due to the diminishing CSA outweighs the strengthening effect, leading to dP becoming negative. For BC with relatively balanced thickness ratios (e.g., 2:1, 1:2, or 1:1), the PIS characterised by the critical strain $\varepsilon_{b}$ demonstrates a significant enhancement compared to Mn8 steel. This improvement is attributed to the synergistic effects of the layered structure, which effectively delays the onset of instability under tensile loading. Plastic instability is observed to initiate more readily in Mn8 steel due to its relatively lower plastic deformation capacity. When Mn8 steel undergoes instability (dP < 0), shear force (τ) develops at the interface with the adjacent SS400 layer. This interfacial shear force arises from the constraint imposed by the more ductile SS400 layer, which compensates by undergoing larger plastic deformation. The additional plasticity of SS400 plays a critical role in resisting the propagation of instability by redistributing the applied stress and delaying catastrophic failure in the BC. As deformation progresses, the capacity of the SS400 layer for micro-deformation becomes exhausted, leading to the propagation of instability throughout the Mn8/SS400 BC. However, measuring and characterising the interfacial shear force (τ) and the micro-deformation of the SS400 steel layer remains challenging.

**Figure 9:**
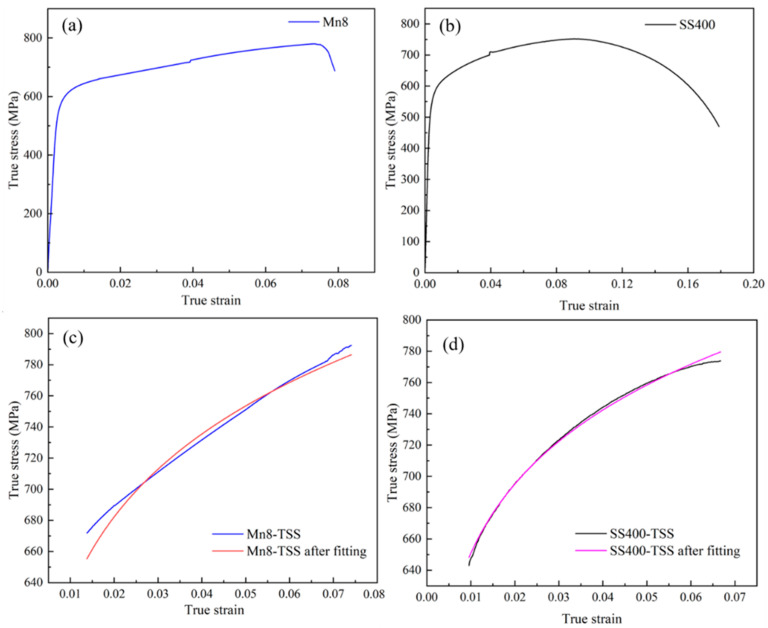
TSS curves with the strain rate transition at 4% strain for (**a**) Mn8 steel and (**b**) SS400 steel; intercepted tensile curves at uniform plastic deformation and their fitted curves for (**c**) Mn8 steel and (**d**) SS400 steel.

Research indicates that PIS is influenced by several factors, including the strain hardening index (n), the work hardening coefficient (k), and the strain rate sensitivity coefficient (m) [50,51]. Accurate prediction of the PIS for Mn8/SS400 BCs requires precise determination of the parameter values for n, k, and m. The strain rate sensitivity coefficient m was evaluated by varying the tensile strain rate at 4% strain for Mn8 and SS400, as shown in Figure 9a,b. The value of m was determined using Equation (6) [52].

(6) $$m=\frac{\ln\left(\sigma_{2}/\sigma_{1}\right)}{\ln\left({\dot{\varepsilon }}_{2}/{\dot{\varepsilon }}_{1}\right)}$$

where $\sigma_{1}$ and $\sigma_{2}$ represent the stresses at a specific strain (4%) corresponding to the varied strain rates; ${\dot{\varepsilon }}_{1}$ denotes the initial strain rate (1 × 10^−4^ s^−1^), and ${\dot{\varepsilon }}_{2}$ indicates the modified strain rate (1 × 10^−3^ s^−1^).

The TSS curves of Mn8 steel (strain range: 0.52%–7.41%) and SS400 steel (strain range: 0.96%–6.67%) were extracted for analysis. The n and k values for Mn8 and SS400 were calculated in conjunction with m using the Hollomon strain-hardening theory [50,51], expressed as follows:

(7) $$\sigma =k\left[\varepsilon^{n}+m\;ln\left(\frac{\dot{\varepsilon }}{\dot{\varepsilon_{r}}}\right)\right]$$

where σ is the stress, ε is the strain, $\dot{\varepsilon }$ is the strain rate, and $\dot{\varepsilon_{r}}$ is the reference strain rate (5 × 10^−5^ s^−1^) [50]. The parameters were determined using the orthogonal distance regression (ODR) fitting method. The intercepted tensile curves at uniform plastic deformation, along with their corresponding fitted curves for Mn8 steel and SS400 steel, are presented in Figure 9c,d. The summarised results are provided in Table 4. The differences between the Mn8 steel (medium manganese, high-carbon austenitic steel) and SS400 steel (low-carbon ferritic-pearlitic steel) in n, k, and m reflect their distinct microstructural behaviours. The comparable n values suggest similar strain-hardening trends, primarily influenced by dislocation dynamics. However, Mn8 steel displays a higher k. This enhanced hardening capacity is attributed to its homogeneous austenitic structure, greater dislocation accumulation, and solid solution strengthening from carbon and manganese. In contrast, the lower m value of Mn8 steel reflects reduced dynamic recovery and diffusional activity. This starkly contrasts SS400, where the ferritic phase demonstrates higher sensitivity, contributing to its relatively higher m value.

Torre et al. [53] mentioned the presence of imperfect regions exhibiting microstructural inhomogeneities within the gauge section of the original tensile specimens. The imperfect region, along with the remaining gauge length (perfect region), undergoes simultaneous plastic deformation, ultimately leading to localised necking and fracture within this region [51]. The parameter η is defined as the ratio of the reduction in the cross-sectional area of the initial imperfect region to the total cross-sectional area of the uniform section:

(8) $$\eta =\frac{A_{0,in}-A_{in}}{A_{0,in}}$$

where $A_{0,in}$ and $A_{in}$ denote the initial cross-sectional areas of the uniform and imperfect sections, respectively. Parameters corresponding to the behaviour of the essentially uniform sections, or equivalently of a perfect section, are denoted with a subscript 0. The deformation within the gauge length section, encompassing both uniform and non-uniform regions, is considered equivalent, and theoretical analysis of such deformation in Mn8/SS400 BCs has been extensively studied using the ROM:

(9) $$\left(V_{Mn8}\sigma_{Mn8}+V_{SS400}\sigma_{SS400}\right)A=\left(V_{Mn8}\sigma_{Mn8,0}+V_{SS400}\sigma_{SS400,0}\right)A_{0}$$

Here, A and $A_{0}$ correspond to the instantaneous cross-sectional areas of the non-uniform and uniform deformation regions, respectively. Under the assumption of constant volume, the local strain values ε and $\varepsilon_{0}$ in the reduced and uniform portions of the gauge section, respectively, are determined as follows:

(10) $$\varepsilon =-ln\frac{A}{A_{in}}$$

(11) $$\varepsilon_{0}=-ln\frac{A_{0}}{A_{0,in}}$$

Substituting Equations (6)–(8), (10) and (11) into Equation (9) leads to Equation (12):

(12) $$\begin{array}{c} e^{-\varepsilon }\left[V_{Mn8}K_{Mn8}\varepsilon^{n_{Mn8}}+V_{SS400}K_{SS400}\varepsilon^{n_{SS400}}+\left(V_{Mn8}K_{Mn8}m_{Mn8}+V_{SS400}K_{SS400}m_{SS400}\right)ln\left(\frac{\dot{\varepsilon }}{\dot{\varepsilon_{r}}}\right)\right]\\ =\frac{e^{-\varepsilon_{0}}}{1-\eta }\left[V_{Mn8}K_{Mn8}\varepsilon_{0}^{n_{Mn8}}+V_{SS400}K_{SS400}\varepsilon_{0}^{n_{SS400}}+\left(V_{Mn8}K_{Mn8}m_{Mn8}+V_{SS400}K_{SS400}m_{SS400}\right)ln\left(\frac{\dot{\varepsilon }}{\dot{\varepsilon_{r}}}\right)\right] \end{array}$$

Here, two dimensionless variables are defined as follows:

(13) $$\beta =\frac{V_{Mn8}K_{Mn8}}{V_{Mn8}K_{Mn8}+V_{SS400}K_{SS400}}$$

(14) $$\mu =\beta m_{Mn8}+\left(1-\beta \right)m_{SS400}$$

The variable β, referred to as the load-sharing coefficient, quantifies the fraction of load carried by Mn8 steel in a composite. The variable μ is known as the strain-rate mixing index. It represents the strain-rate contributions of the constituent steels within the composite, weighting each by their respective load-sharing fractions (β, 1−β).

Incorporating Equations (13) and (14) into Equation (12), Equation (15) is obtained as follows:

(15) $$ln\left(\frac{\dot{\varepsilon }}{\dot{\varepsilon_{r}}}\right)=\frac{e^{\varepsilon -\varepsilon_{0}}}{1-\eta }\left[\frac{\beta \varepsilon_{0}^{n_{Mn8}}+\left(1-\beta \right)\varepsilon_{0}^{n_{SS400}}}{\mu }+ln\left(\frac{\dot{\varepsilon }}{\dot{\varepsilon_{r}}}\right)\right]-\frac{\beta \varepsilon^{n_{Mn8}}+\left(1-\beta \right)\varepsilon^{n_{SS400}}}{\mu }$$

Equation (15) defines the relationship governing the instantaneous strain distribution between the uniform and non-uniform sections of the composite by consolidating six mechanical property parameters ($V_{Mn8},\;V_{SS400},\;K_{Mn8},\;K_{SS400},\;m_{Mn8},\;m_{SS400})$ into only β and μ, which is also referred to as the long-wavelength approach (LWA). It retains significant theoretical and practical relevance, even in cases where non-uniform strain ($\varepsilon_{0}$) is absent or negligible. Thus, it is employed to predict the PIS of Mn8/SS400 BCs across varying Mn8 volume fractions. The corresponding calculated PIS results and the relative deviation between experimental and predicted PIS values are presented in Table 5. The LWA model predictions align most closely with experimental values when the proportions of Mn8 steel and SS400 steel are balanced. However, when the proportion of Mn8 steel is significantly reduced, the deviation between predicted and experimental values becomes notably larger. Conversely, increasing the proportion of Mn8 steel results in smaller deviations, though discrepancies persist. In fact, the long-wavelength prediction model (Equation (15)) accounts solely for the strain-hardening characteristics of the two layers while overlooking the shear stress transfer arising from the differential deformation behaviour between the materials [25].

### 3.5. The Effect of Strain Hardening Capacity and the Thickness Ratio on Accumulated Damage and Fracture Mechanisms

**Figure 10:**
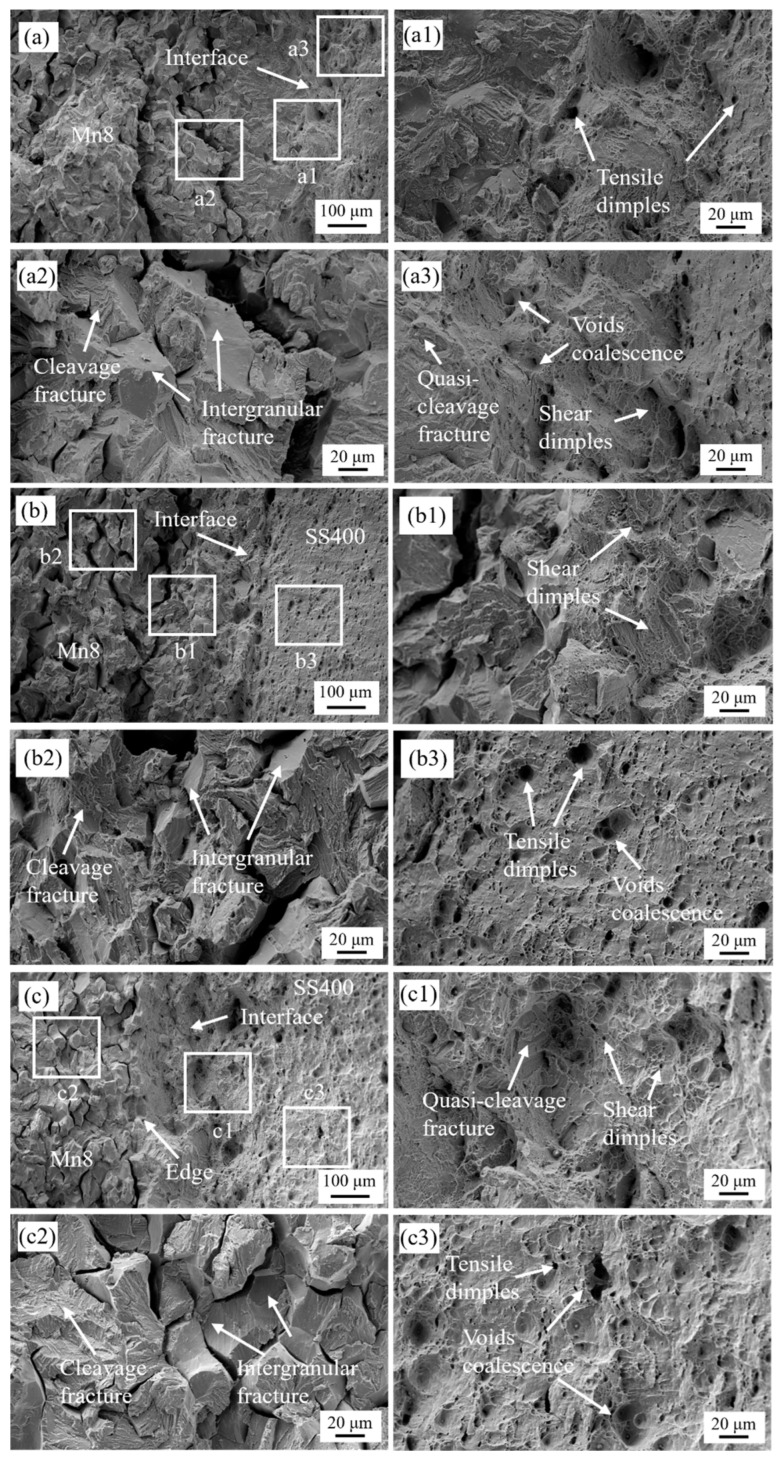
The fracture surfaces of the selected BCs of (**a**) Mn8/SS400 BC-1, (**b**) Mn8/SS400 BC-2, and (**c**) Mn8/SS400 BC-5; (**a1**–**a3**,**b1**–**b3**,**c1**–**c3**) are corresponding magnified views, respectively.

Figure 10 depicts the fracture surfaces of Mn8/SS400 BCs with Mn8 steel layer proportions of 83%, 67%, and 17%. The absence of visible interface cracking or debonding across all configurations indicates that the deformation and fracture of the Mn8 and SS400 layers occur concurrently. Distinct fracture types were observed between the Mn8 and SS400 layers. For Mn8/SS400-1, characterised by an 83% Mn8 layer, large tensile dimples dominate the interface region (Figure 10(a1)), with small shear dimples particularly evident near the interface adjacent to the SS400 layer (Figure 10(a1,a3)). These features align with the characteristics of shear fracture. Furthermore, small dimples within the large tensile dimples (Figure 10(a3)) suggest void coalescence as a key fracture mechanism. By contrast, the fracture surface in Figure 10(a2) exhibits intergranular fracture along prior austenite grain boundaries, driven by impurity segregation at grain boundaries [54,55]. The intergranular fracture results in smooth facets delineating prior austenite grains and secondary cracks perpendicular to the fracture surface, highlighting grain boundary brittleness. Intergranular fracture regions are interspersed with cleavage fracture zones, characterised by river patterns and facet steps, indicating competition between the intergranular and transgranular fractures. The lower UTS of Mn8/SS400-1 is predominantly attributed to the brittle behaviour of the Mn8 steel layer, which constitutes the majority of the composite and lacks sufficient ductility. Stress concentration at the interface, coupled with the limited toughening effect of the SS400 layer, accelerates crack propagation and precipitates early tensile failure.

For Mn8/SS400-2, with a 2:1 Mn8-to-SS400 ratio, the interface exhibits downward sinking as the origin of failure, forming slanted edges around the sample (Figure 10b). Shear dimples are prominent near the interface (Figure 10(b1)), while tensile dimples and void coalescence dominate the SS400 layer (Figure 10(b3)). The Mn8 layer maintains a mixed fracture mode, combining intergranular and transgranular features. Notably, the central shrinking zone aligns with the interface rather than the geometric centre, suggesting abnormal slip behaviour. This ratio achieves an optimal balance of brittle and ductile characteristics, with the Mn8 layer providing strength and the SS400 layer delaying crack propagation and absorbing energy. The interface enhances stress distribution, maximising UTS in this configuration. For Mn8/SS400-3 and -4, similar fracture mechanisms are observed. In Mn8/SS400-5, the load borne by the thin Mn8 layer becomes negligible relative to the SS400 layer, contributing minimally to the overall fracture process. The central shrinking zone of necking appears in the SS400 steel layer, with tensile dimples and large void coalescence zones visible at the interface (Figure 10(c1)). This indicates a transformation in fracture mechanisms. Compared to the 2:1 configuration, the UTS decreases as the diminished contribution of Mn8’s high strength is not fully offset by the SS400 layer, which, despite enhancing ductility, lacks sufficient strength to sustain higher tensile loads.

### 3.6. Bending Characteristics of the Mn8/SS400 BCs

Because the BC is often prone to nucleate the crack under complex stress, three-point bending tests of different samples were performed to investigate the bending characteristics and crack growth behaviour.

**Figure 11:**
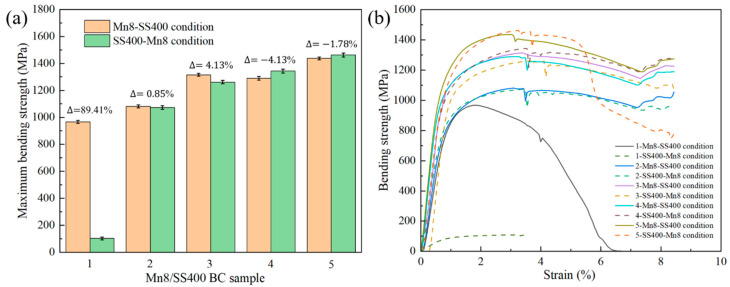
(**a**) Variation in bending strength with strain, and (**b**) histogram of bending strength for Mn8/SS400 BCs with different bending ways.

Figure 11 reveals the influence of thickness ratios on the bending strength of Mn8/SS400 BCs in different bending ways. In contrast to the tensile strength results presented in Figure 4, the bending strength of Mn8/SS400 BCs exhibits a positive correlation with the proportion of SS400 low-carbon steel, as shown in Figure 11a. The maximum bending strength is achieved at 1462.21 MPa when the SS400 content reaches 83% in the SS400-Mn8 condition. Furthermore, the differences in maximum bending strength between different bending ways are generally minimal, except for sample 1 (83% Mn8 steel), where the difference is pronounced, reaching a peak value of 89.41%. Notably, when the Mn8 steel proportion exceeds 50%, the maximum bending strength under the Mn8-SS400 condition surpasses that of the SS400-Mn8 condition. Conversely, for Mn8 steel proportions below 50%, the maximum bending strength under the Mn8-SS400 condition becomes slightly lower than that of the SS400-Mn8 condition.

The bending strength-strain curve in Figure 11b indicates the higher bending strength for the BCs with a larger volume fraction of SS400 steel in the whole deflection, which can be attributed to the enhanced ductility and toughness provided by SS400, which allows the composite to absorb more deformation energy during bending, thereby reducing stress concentration and delaying fracture. Furthermore, the interface between Mn8 and SS400 plays a critical role in the mechanical performance of the composite. Under external stress, the interface acts as a stress transition zone, facilitating stress redistribution across the entire structure. During the bending process, interfacial diffusion contributes to the effective transfer of tensile stress, promoting better load-sharing between the Mn8 and SS400 layers. This synergistic interaction between the high strength of Mn8 steel, the high ductility of SS400, and the stress transfer capability at the interface results in an optimised mechanical response, significantly improving the bending strength of the BCs. Some fluctuations in the curve are mostly ascribed to the slip between the sample and supporting pins because of the large deflection [56].

**Figure 12:**
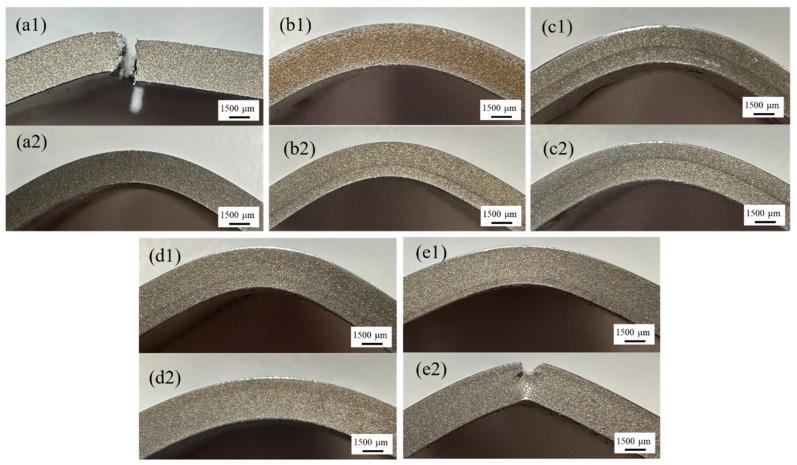
Macrographs of Mn8/SS400 BCs after bending tests under different bending ways: (**a1**–**e1**) Mn8-SS400 condition and (**a2**–**e2**) SS400-Mn8 condition, corresponding to Mn8/SS400 BCs-1 to BCs-5.

Additionally, the bending curves of Mn8/SS400 BC samples 2, 3, and 4 under both bending conditions and sample 5 in the Mn8-SS400 condition exhibit a consistent trend where the strength decreases gradually as the extension reaches 30 mm. As shown in Figure 12(b1–d1,b2–d2), no cracks or fractures occur under either bending way. This behaviour can be attributed to the balanced stress distribution and the synergistic interaction between the high-strength Mn8 steel and the ductile SS400 steel, which enables effective load sharing between the two layers. Furthermore, the neutral axis shifts optimally depending on the thickness ratio, ensuring that excessive stress is avoided in either layer, thereby enhancing the composite’s resistance to crack initiation and propagation. In contrast, sample 1 under the Mn8-SS400 condition and sample 5 under the SS400-Mn8 condition display multiple progressive load drops, indicating cracking events occurring within the composite layers, as shown in Figure 12(a1,a2). The significant stress drop observed in sample 1 under the Mn8-SS400 condition is primarily attributed to the catastrophic fracture of the composite. When Mn8 steel constitutes 83% of the composite and is positioned on the loading side (Mn8-SS400), the brittle nature of Mn8 steel dominates the mechanical response. The reduced proportion of SS400 limits its ability to provide ductile support, while the interface loses its capacity to resist crack propagation effectively. Consequently, cracks initiate and propagate rapidly through the Mn8 steel layer, resulting in the complete failure of the composite plate. In contrast, under the SS400-Mn8 condition, the composite remains intact without visible cracks. This behaviour arises because the tensile stress on the tension side is more critical than the compressive stress on the compression side, as most materials exhibit lower tensile strength compared to compressive strength. SS400 steel, being a ductile material, effectively absorbs compressive stress through plastic deformation, redistributing the load and delaying failure. Furthermore, in sample 5 under the SS400-Mn8 condition, the observed stress decline is associated with progressive crack propagation within the Mn8 layer, as shown in Figure 12(e2). When Mn8 steel accounts for 17% of the composite and the loading point is on the SS400 side, cracks initiate in the brittle Mn8 steel layer but are gradually arrested as they approach the ductile SS400 layer. The SS400 layer, with its high ductility, suppresses further crack growth through an intrinsic toughening mechanism by absorbing deformation energy and redistributing stress. The interface further delays crack propagation beyond the Mn8 layer, preventing complete failure of the composite. This toughening mechanism, driven by the high ductility and energy absorption capacity of the SS400 layer, enhances the overall fracture resistance of the composite and effectively delays catastrophic failure.

Material toughness, representing the ability to absorb energy before failure, is directly reflected in the area under the bending strength–strain curve. For BCs with a higher volume fraction of SS400, this area is significantly larger, indicating superior energy absorption capacity during bending due to the high ductility and toughness of the SS400 layer. This enhanced toughness demonstrates that BCs with a larger SS400 fraction are better equipped to withstand bending loads without catastrophic failure. Interestingly, this trend contrasts with tensile test results, where the UTS increases as the thickness ratio between the Mn8 steel and SS400 becomes balanced. The discrepancy arises from the distinct stress distribution and deformation mechanisms involved in bending and tensile loading. Under bending, the ductile SS400 layer primarily governs strain accommodation and crack arrest, enhancing the composite’s toughness. Conversely, in tensile loading, the balanced configuration optimises the synergy between the high strength of Mn8 steel and the moderate ductility of SS400, maximising UTS. These findings underscore the critical role of layer composition and loading conditions in tailoring the mechanical performance of composite materials for specific applications.

## 4. Conclusions

This study systematically investigated the mechanical behaviour of Mn8/SS400 BCs with different thickness ratios through uniaxial tensile testing. Comprehensive analyses of UTS, PIS, and fracture mechanisms led to the following conclusions:

(1) The UTS and elongation of the Mn8/SS400 BCs exhibit a non-linear relationship with varying thickness ratios. Intermediate ratios (e.g., 2:1) maximise strength and ductility due to optimised load transfer and balanced deformation compatibility between the layers. Extreme ratios (1:5 and 5:1) result in reduced mechanical performance due to mismatched strain hardening and interfacial stress concentration.
(2) The fracture behaviour transitions from intergranular and transgranular fractures in Mn8-rich BCs to ductile dimples and void coalescence in SS400-dominant composites. At intermediate ratios, the synergistic interaction between brittle Mn8 and ductile SS400 layers delays crack propagation and enhances stress distribution, leading to superior fracture resistance.
(3) Enhanced strain hardening near the interface, driven by dislocation pile-ups and diffusion strengthening, suppresses premature instability in BCs with balanced thickness ratios. However, excessive layer thickness disparities compromise strain compatibility, accelerating instability and reducing overall mechanical performance.
(4) The diffusion layer at the interface of Mn8/SS400 BCs and the resulting high dislocation density enhance the interfacial bonding strength. This mechanism, combined with stress redistribution, enables effective strain transfer, contributing to the superior performance of the BCs.

## Figures and Tables

**Table 1:** Chemical compositions of Mn8 and SS400 layers of BC (wt.%).

Material	C	Si	Mn	Cr	Mo	V	Ti	P	S
Mn8	0.98	0.19	8.10	1.39	0.26	0.18	0.05	0.012	0.002
SS400	0.085	0.28	1.43	-	-	-	0.012	0.013	0.001

**Table 2:** The categorisation of Mn8/SS400 BC samples.

Sample	Mn8 Fraction (%)
Mn8	100
Mn8/SS400-1	83
Mn8/SS400-2	67
Mn8/SS400-3	50
Mn8/SS400-4	33
Mn8/SS400-5	17
SS400	0

**Table 3:** YS and UTS of Mn8/SS400 BCs.

	Yield Strength (MPa)	Ultimate Tensile Strength (MPa)	Ultimate Elongation (%)
Mn8/SS400-Mn8	597.96 ± 4.16	792.68 ± 5.33	7.43 ± 1.33
Mn8/SS400-1	587.35 ± 5.27	779.20 ± 7.24	5.49 ± 0.63
Mn8/SS400-2	620.03 ± 3.64	938.19 ± 6.80	14.91 ± 3.27
Mn8/SS400-3	556.59 ± 6.11	838.71 ± 5.83	12.23 ± 2.67
Mn8/SS400-4	604.14 ± 5.48	881.50 ± 5.66	13.54 ± 1.94
Mn8/SS400-5	583.45 ± 7.03	795.69 ± 4.79	4.92 ± 0.51
Mn8/SS400-SS400	542.70 ± 6.21	774.19 ± 6.43	12.34 ± 2.54

**Table 4:** Strain hardening index (n), work hardening coefficient (k), and strain rate sensitivity coefficient (m) for Mn8 and SS400 steel.

	n	k	m
Mn8	0.11 ± 0.0020	1024.03 ± 1.72	4.32 × 10^−3^
SS400	0.10 ± 0.0013	989.93 ± 1.03	8.6 × 10^−3^

**Table 5:** Comparison of experimental and predicted PIS with relative deviation.

	Exp. of PIS/%	Pre. of PIS by Equation (12)	Deviation from Exp./%
Mn8	7.43	8.87	16.2
Mn8/SS400-1	5.49	6.73	18.4
Mn8/SS400-2	14.91	16.15	7.7
Mn8/SS400-3	12.23	14.41	15.1
Mn8/SS400-4	13.54	15.49	12.6
Mn8/SS400-5	4.92	6.64	25.9
SS400	12.34	18.93	34.8

## Data Availability

The data that support the findings will be available in FAIRsharing.org: MDF; The Materials Data Facility at 10.25504/FAIRsharing.I5xHsJ following an embargo from the date of publication to allow for commercialization of research findings.
